# Vestibular Evoked Myogenic Potentials to Diagnose Vestibular Neuritis: A Scoping Review

**DOI:** 10.1002/lary.70214

**Published:** 2025-10-22

**Authors:** Diego Piatti, Laura Casagrande Conti, Gianluca Paolocci, Iole Indovina, Marco Tramontano, Leonardo Manzari

**Affiliations:** ^1^ Laboratory of Neuromotor Physiology, Santa Lucia Foundation Scientific Institute for Research and Health Care Rome Italy; ^2^ Department of Biomedical and Dental Sciences and Morphofunctional Imaging University of Messina Messina Italy; ^3^ Department of Systems Medicine and Centre for Space BioMedicine University of Rome Tor Vergata Rome Italy; ^4^ Department of Biomedical and Neuromotor Sciences University of Bologna Bologna Italy; ^5^ Unit of Occupational Medicine IRCCS Azienda Ospedaliero‐Universitaria di Bologna Bologna Italy; ^6^ MSA ENT Academy Center Cassino Italy

**Keywords:** balance, VEMPS, vestibular neuritis, vestibular rehabilitation, vestibular system

## Abstract

**Objective:**

This scoping review aims to explore the diagnostic value of Vestibular Evoked Myogenic Potentials (VEMPs) in people with Vestibular Neuritis (VN) with a specific focus on the differential contribution of ocular (oVEMPs) and cervical (cVEMPs) recordings.

**Data Sources:**

A comprehensive search was conducted in December 2024 across PubMed, Scopus, and the Cochrane Library. Studies published in English and involving adult patients with suspected or confirmed VN and assessing VEMPs were considered eligible.

**Review Methods:**

The PRISMA‐ScR guidelines were followed. Data were extracted regarding methodology, patient characteristics, and diagnostic performance of VEMPs.

**Results:**

Eighteen studies met all eligibility criteria. Included studies consistently demonstrated that VEMPs offer valuable diagnostic information in VN, particularly in identifying the affected branch of the vestibular nerve. Several studies supported the use of VEMPs in combination with other vestibular tests to enhance diagnostic accuracy. Methodological heterogeneity in stimulation parameters and recording techniques limits direct comparison across studies but underscores the need for protocol standardization.

**Conclusion:**

VEMPs are a useful adjunctive tool in the diagnosis of VN, particularly for topographic localization of vestibular nerve involvement. Future research should focus on standardizing protocols and exploring correlations with clinical outcomes to refine their diagnostic utility further.

## Graphical abstract

Standardization of stimulation and recording protocols is essential to improve comparability across VEMP studies. Integrating cervical and ocular VEMPs with other vestibular tests enhances diagnostic accuracy and supports precise localization of vestibular neuritis.

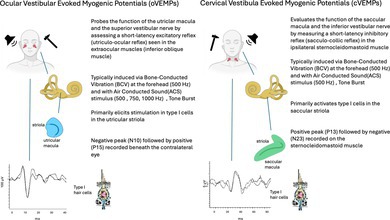

## 1 Introduction

Vestibular neuritis (VN) is an acute otoneurological disorder characterized by the sudden onset of prolonged vertigo of peripheral origin. It is considered one of the most common causes of acute vestibular syndrome (AVS) [1]. The typical clinical picture includes intense rotatory vertigo, oscillopsia, postural imbalance with a tendency to fall toward the affected side, nausea, vomiting, and the absence of cochlear symptoms, such as hearing loss or tinnitus, distinguishing it from other peripheral vestibulopathies like labyrinthitis [2]. Given the significant symptomatic overlap between peripheral vestibular disorders and central causes, particularly cerebellar and brainstem stroke, accurate and early differential diagnosis is crucial, especially in emergency settings.

People with vestibular neuritis (PwVN) often present with static and dynamic clinical signs. Among the static signs, we recognize: spontaneous horizontal‐torsional nystagmus usually beating away from the affected side with the horizontal component, abnormal ocular counter‐rolling—the eyes may rotate toward the healthy ear side, reflecting the imbalance in otolith signals, head tilting, and skewing toward the affected side with skew deviation, and finally, abnormal Subjective Visual Vertical (SVV) [3].

Among the dynamic signs we recognize: a positive head impulse test (HIT) or video head impulse test (vHIT) [4], or otolith instrumental dynamic function signs such as early (n10 and p13‐23) complexes in evoked ocular and cervical vestibular myogenic potentials. Alterations in these evoked myogenic potentials (VEMPs) are characterized by pathological variation of the Asymmetry Ratio, showing a difference in VEMPs amplitude between the two ears [5, 6, 7]. Specifically, the asymmetry ratio is calculated by comparing the peak‐to‐peak amplitude of the n10 component in ocular VEMPs (oVEMPs) and the p13–n23 complex in cervical VEMPs (cVEMPs) between the suspected affected ear and the contralateral (unaffected) ear [3, 7].

In the mammalian vestibular system, the otolith organs serve as the sensors for linear motion. Otolith afferents are divided into two distinct functional pathways. The so‐called transient (or dynamic) system is especially responsive to rapid changes in linear acceleration, also known as “jerks.” It receives input from highly specialized type I vestibular hair cells located in the striolar region, which are connected to postsynaptic vestibular afferents with irregular resting activity through ultrafast calyx‐type synapses [8]. In contrast, constant or low‐frequency linear accelerations are processed by the sustained (or static) pathway. This system primarily involves type II vestibular hair cells and regular vestibular afferents originating from the extrastriolar region.

To study dynamic otolith function, two main approaches have been employed: eccentric displacement (unilateral stimulation achieved by moving a subject's chair radially away from the vertical rotation axis at a constant angular velocity) and tilt mode (bilateral stimulation by tilting the entire head and body about the naso‐occipital axis). Although these methods have produced valuable research data, they are not suitable for routine clinical practice.

As a result, the dynamic functional assessment of the otolith organs was not easily accessible in clinical settings until approximately 30 years ago, when two important clinical tests were introduced. The first is cVEMPs, recorded from contracted sternocleidomastoid (SCM) muscles in response to air‐conducted sound (ACS) [9] or bone‐conducted vibration (BCV) [10]. The second is the oVEMPs in response to BCV [11, 12].

Since then, numerous types of VEMPs have been described, reflecting the widespread projection of vestibular afferent input, via the vestibular nuclei, to various muscle groups. The oVEMPs measure myogenic potentials from the inferior oblique (IO) eye muscle, while the cVEMPs measure potentials from the tensed sternocleidomastoid (SCM) neck muscle. Although both VEMPs are elicited by similar stimuli, sound and vibration, they exhibit distinct characteristics.

The first negative component of the oVEMPs, known as n10, is a small (~5–10 μV) excitatory potential appearing approximately 10 ms after stimulus onset, recorded via surface EMG electrodes positioned just below the eyes (IO muscle) while the subject looks upward. This response is a crossed potential, originating primarily from utricular afferents traveling through the superior division of the vestibular nerve contralateral to the excited ocular muscle (IO) [13].

In contrast, the first positive component of the cVEMPs, referred to as p13‐n23, is an uncrossed inhibitory potential recorded over the SCM while the subject lifts or turns their head. It primarily originates from saccular afferents carried by the inferior vestibular nerve to the inhibited ipsilateral SCM [14].

The vHIT and caloric testing represent core diagnostic tools for VN, although they are tests that evaluate the function of the semicircular canals only, while other assessments, such as VEMPs and SVV, are considered supportive [1]. Among these, VEMPs have gained attention for their ability to evaluate otolith organ function and to contribute to the topographic classification of vestibular nerve involvement.

Specifically, oVEMPs offer a non‐invasive means of evaluating utricular and superior vestibular nerve function [15], and are typically reduced or absent in superior and complete AUVP [16]. cVEMPs reflect saccular and inferior vestibular nerve function and are diminished in cases involving inferior nerve involvement [1].

Despite the increasing clinical use of VEMPs, their specific utility in VN diagnosis remains under‐investigated. While individual studies have explored their sensitivity and topographic value, no review has yet evaluated the clinical value in diagnosing VN.

Therefore, the aim of this scoping review is to explore the diagnostic contribution of both cVEMPs and oVEMPs in VN diagnosis.

## 2 Methods

This scoping review was reported in accordance with the Preferred Reporting Items for Systematic Reviews and Meta‐Analyses extension for Scoping Reviews (PRISMA‐ScR) guidelines [17] and followed the methodological recommendations outlined in the Cochrane Handbook for Systematic Reviews of Interventions [18].

### 2.1 Search Strategy and Eligibility Criteria

A comprehensive search strategy was applied in December 2024 across the electronic databases PubMed, Scopus, and the Cochrane Library. The following search terms were used:

(“vestibular neuritis”[MeSH Terms] OR “vestibular neuritis” OR “vestibular neuronitis”) AND (“vestibular evoked myogenic potentials”[MeSH Terms] OR “VEMPs” OR “cVEMPs” OR “oVEMPs” OR “vestibular myogenic potentials”) AND (“diagnosis”[MeSH Terms] OR “diagnostic” OR “evaluation”). Search terms were adapted to the indexing system of each database, and appropriate filters and subheadings were applied. The detailed search strategy is provided in Supporting Information S1.

Only peer‐reviewed articles published from inception to December 20, 2024 were considered. Eligible study designs included randomized controlled trials, cohort studies, case–control studies, and cross‐sectional studies. Case reports, reviews, conference abstracts, and study protocols were excluded.

Inclusion criteria consisted of studies involving adults (≥ 18 years) with a diagnosis or suspicion of VN; studies that assessed VEMPs; and articles available in full text and written in English. Exclusion criteria included studies addressing conditions other than vestibular neuritis and studies evaluating diagnostic tools other than VEMPs.

### 2.2 Study Selection and Data Collection Process

All identified studies were uploaded to the Rayyan web‐based platform for review screening [19]. Screening included title and abstract evaluation, with final full‐text assessment.

Screening and data extraction were independently performed by two reviewers (DP and LCC). In the case of disagreement, a third reviewer (MT) was consulted to reach consensus. Data were extracted regarding study design, methodology, participant characteristics, diagnostic criteria, and outcomes related to VEMPs and were recorded in a structured Excel spreadsheet.

The entire process was conducted according to PRISMA‐ScR guidelines [17]. The PRISMA‐ScR checklist is available in Supporting Information S2.

### 2.3 PICO Question

The research question was developed according to the PICO framework [20] to define the review's objectives and guide the search strategy. The population (P) included individuals diagnosed with VN; the intervention (I) was the assessment through VEMPs; the comparator (C) referred to other vestibular function tests; the outcome (O) was not applicable, as the review focused on diagnostic contribution rather than clinical effectiveness. The aim was to provide a structured response to the question: What is the role of VEMPs in the diagnosis of VN?

## 3 Results

### 3.1 Study Selection

**FIGURE 1:**
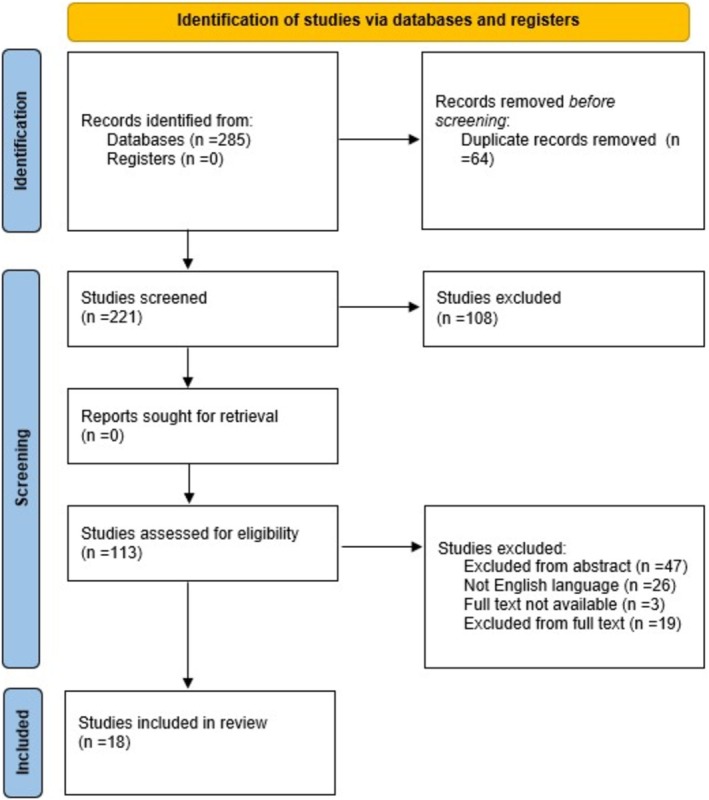
The flow chart detailing the article screening process. Following the application of inclusion and exclusion criteria, a total of 18 articles were ultimately included in the review. [Color figure can be viewed in the online issue, which is available at www.laryngoscope.com]

Initially, 285 records were identified across the three electronic databases. After removing 64 duplicates, 221 articles remained. Title screening resulted in the exclusion of 108 studies and the retention of 113. Abstract screening of these 113 articles led to the inclusion of 66 and the exclusion of 47. Full‐text screening was then performed on the 66 retained articles. Of these, 26 were not published in English, 3 full texts were unavailable, and 16 were excluded after full‐text evaluation due to not meeting the inclusion criteria. When possible, in an attempt to retrieve the unavailable full texts, we tried to contact the authors via e‐mail or through (https://www.researchgate.net/). Furthermore, three reviews were excluded because they did not meet the inclusion criteria of our scoping review; however, they provide a preliminary insight into the utility and potential need for VEMP testing in VN [21, 22, 23]. Ultimately, 18 studies met all eligibility criteria and were included in the final analysis. The PRISMA flow diagram is reported in Figure 1 [17].

The study selection process was independently conducted by two reviewers in a blinded manner (DP and LCC). In the event of disagreement, a third reviewer (MT) was consulted to resolve conflicts following the unblinding of the initial reviewers.

### 3.2 Study Characteristics

An initial classification of the included studies by design was performed. The majority of the studies adopted a cross‐sectional design [16, 24, 25, 26, 27, 28, 29, 30, 31, 32]. A smaller group of studies employed prospective designs [33, 34, 35, 36, 37], while others were retrospective in nature [38, 39, 40].

Across the included studies, a common methodological feature was the comparison of VEMPs results in PwVN against either established normative values or healthy control groups. This consistent approach facilitated the evaluation of the diagnostic utility of VEMPs within a clinical population.

### 3.3 Results of Individual Studies

Several studies have investigated the diagnostic contribution of VEMPs in patients with VN, focusing on both methodological aspects and clinical interpretation of findings.

Electrode placement and recording conditions varied across studies. It is crucial to emphasize that successful cVEMPs acquisition relies on a tonic contraction of the SCM and the IO muscle; for this reason, many protocols recommend testing patients in a supine position with the head actively rotated and the eyes looking at a point placed high up, slightly behind the position of the head [5]. Hong and colleagues [24] recorded cVEMPs in seated patients with the head rotated away from the stimulated side. The active electrode was placed over the middle third of the sternocleidomastoid muscle, the reference on the upper sternum, and the ground on the forehead. In this cohort of 134 PwVN, 36.6% showed abnormal VEMPs responses when age‐adjusted normative values were applied, highlighting the importance of considering age‐related changes.

Manzari and colleagues [37] examined the n10 component of oVEMPs in 133 patients with unilateral superior vestibular neuritis and preserved inferior vestibular nerve function in the acute stage of the disease. Recordings were obtained in the supine position with electrodes placed near the lower eyelid and 2 cm below. By applying BCV at Fz to stimulate both labyrinths simultaneously in people with superior vestibular neuritis (SVN), they demonstrated a dissociation between two measures of otolithic function in the affected ear. This study confirmed that the n10 component of oVEMPs in response to BCV is mediated primarily by utricular receptors via the superior vestibular nerve, emphasizing that the test battery must be comprehensive, with ocular and cervical VEMPs being complementary to each other.

Curthoys [16], Oh and colleagues [27] similarly demonstrated that the n10 response of oVEMPs to ACS or BCV depends on the integrity of the superior vestibular nerve, reflecting utricular afferents. Another study [25] further supported this distinction, reporting that loss of ACS‐evoked oVEMPs with preserved cVEMPs is typical of superior nerve involvement in PwVN.

Several studies emphasized the clinical utility of combining VEMPs with other vestibular tests [26, 28, 34, 35]. Lin and colleagues [26] used audiometry, caloric testing, cVEMPs, and oVEMPs, and concluded that this integrated approach enhances the identification of the affected nerve branch and may aid in predicting symptom resolution. Two studies [28, 34] proposed that VEMPs testing can be a useful adjunct to vHIT in both diagnosis and monitoring of VN. Magliulo and colleagues [35] also endorsed the use of VEMPs and vHIT for identifying selective vestibular nerve damage. The study of Skorić and colleagues [38] found a significant correlation between oVEMPs responses and vHIT asymmetry, supporting the combined application of both tools.

Viciana and colleagues [33] observed prolonged ipsilateral latencies for P1 and N1 peaks in cVEMPs, with occasional contralateral delay. However, no significant correlation was found between VEMPs and self‐reported quality‐of‐life outcomes as measured by the SF‐36 or DHI. Notably, inferior vestibular nerve involvement was identified in approximately half of the patients. The main features of the various studies we analyzed are summarized in Table S1.

The key characteristics of each included observational study, including study design, sample size, type of VEMPs tested (cVEMPs, oVEMPs, or both), stimulation modality, recording conditions, and main findings are reported in Table 1.

**TABLE 1:** Stimulation parameters and corresponding results summarized.

Author, year	N VN patients	Type of VEMPS	Sound conduction	Stimuli	Hz	Significant findings in VEMPs responses
Hong, 2008	134 patients	cVEMPs	NA	Clicks	NA	36.6%
Manzari, 2010	133 patients	oVEMPs cVEMPs	BCV ACS	Tone burst Tone burst	500 Hz 500 Hz	94%
Viciana, 2010	25 patients	cVEMPs	ACS	Tone burst	500 Hz	51%
Curthoys, 2011	10 patients	oVEMPs cVEMPs	ACS BCV	Tone burst	500 Hz	ACS cVEMPs: 0.50% ACS oVEMPs: 55.49% BCV oVEMPs: 51.63%
Govender, 2011	23 patients	oVEMPs cVEMPs	ACS BCV	Tone burst	400–500 Hz	The abolition of the oVEMPs indicates involvement of the superior part of the nerve, thus loss of the AC evoked oVEMPs with preservation of the AC evoked cVEMPs are the usual findings in pwVN
Lin, 2011	20 patients	oVEMPs cVEMPs	ACS BCV	Tone burst	5 Hz 5 Hz	50% 25%
Oh, 2013	30 patients	oVEMPs cVEMPs	ACS BCV	Tone burst	1000 Hz 500 Hz	ACS cVEMPs: 27.4% ± 25.8% BCV cVEMPs: 27.9% ± 18.0% ACS oVEMPs: 52.0% ± 39.5 BCV oVEMPs middle forehead: 41.9% ± 33.9% BCV oVEMPs mastoid process: 52.44% ± 39.3%
Walther, 2013	20 patients	oVEMPs cVEMPs	ACS	Tone burst	500 Hz	The use of vHIT together with oVEMPs and cVEMPs to ACS allows a more precise differential topologic
Adamec, 2013	26 patients	oVEMPs cVEMPs	ACS	Clicks	NA	Prolonged latencies of oVEMPs were associated with oVEMPs recovery on follow‐up
Magliulo, 2014	28 patients	oVEMPs cVEMPs	BCV ACS	NA	500 Hz	28.5%
Magliulo, 2014	40 patients	oVEMPs cVEMPs	BCV ACS	NA	500 Hz	oVEMPs 80% cVEMPs 47%
Nagai, 2014	22 patients	oVEMPs cVEMPs	BCV	Clicks	500 Hz	oVEMP 68.2% cVEMP 27%
Skorić, 2017	31 patients	oVEMPs cVEMPs	ACS	Clicks	NA	oVEMPs 80%
Cherchi, 2019	15 patients	oVEMPs	NA	NA	NA	oVEMPs 87%
Tripathi, 2020	10 patients	cVEMPs	ACS	Tone burst	500 Hz	3%
Calic, 2020	22 patients	oVEMPs cVEMPs	ACS + BCV	Clicks	NA	The absent or asymmetric oVEMPs was a relatively insensitive (50%) but specific (90.9%) marker of VN. Similar rates of abnormality of cVEMP in VN and PCS indicate that this test is a poor discriminator
Manzari, 2021	39 patients	oVEMPs	ACS BCV	NA	500 Hz	All patients showed a significant alteration (asymmetry ratio more than 40%) for ocular vestibular‐evoked myogenic potentials
Kabis, 2022	37 patients	oVEMPs cVEMPs	NA	Clicks	NA	oVEMPs: 54% cVEMPs: 43%

## 4 Discussion

**FIGURE 2:**
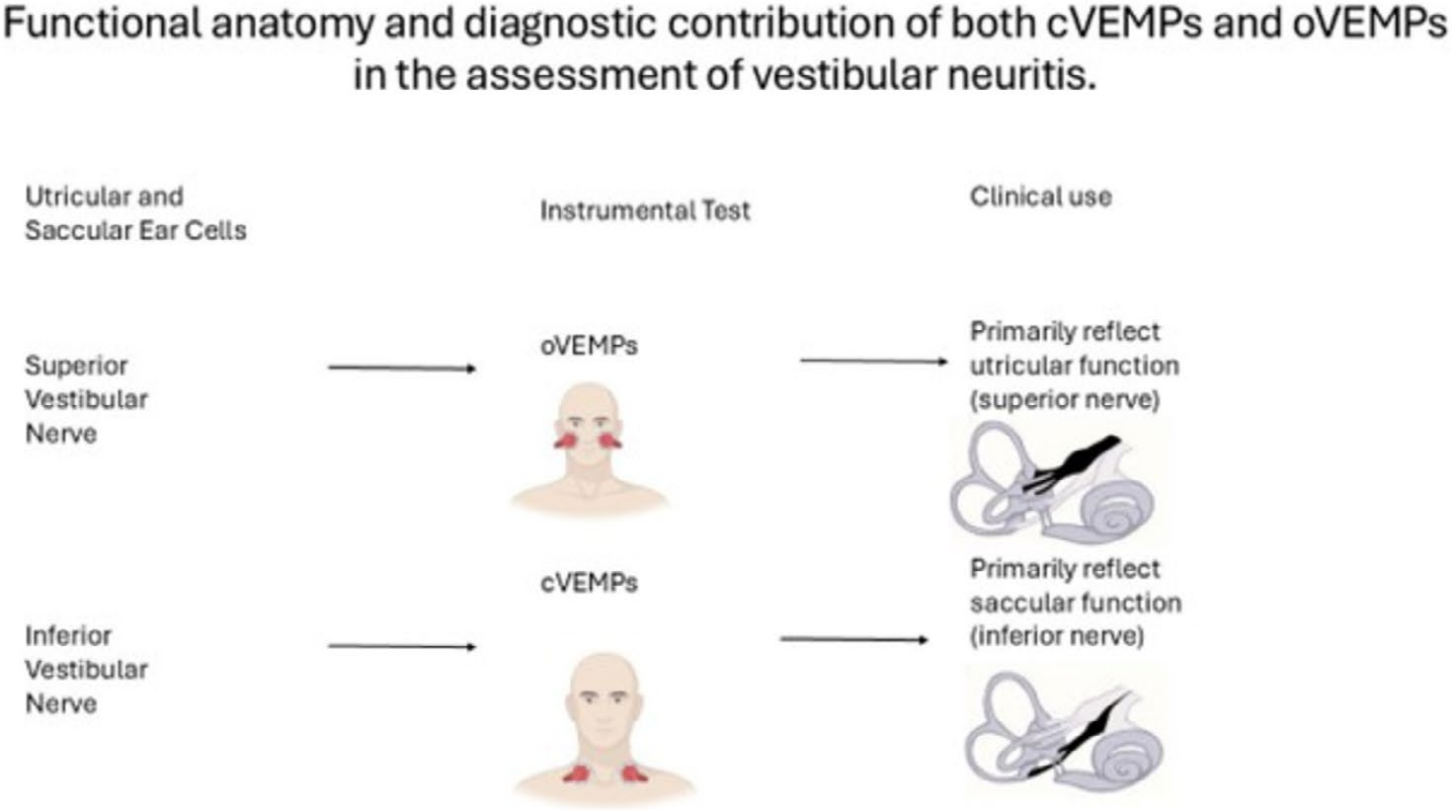
The clinical contribution of oVEMPs and cVEMPs. Part of the figure was adapted from [41]. [Color figure can be viewed in the online issue, which is available at www.laryngoscope.com]

The aim of this scoping review was to explore the role of the VEMPs in diagnosing VN. Although VEMPs have demonstrated clear diagnostic value, it is important to recognize that they assess specific aspects of vestibular function: oVEMPs and superior vestibular nerve function while cVEMPs assess saccular and inferior vestibular nerve function [31, 37, 41] as reported in Figure 2. Therefore, their clinical application should be driven by a clear rationale based on symptomatology and the suspected topographic localization of the lesion.

VEMPs testing has emerged as a useful diagnostic tool, providing functional insights into otolith organ integrity. The application of VEMPs evoked by sound and vibration in vestibular diagnostics is supported by robust evidence [42]: (1) control data from patients lacking vestibular function; (2) neural evidence demonstrating that these stimuli indeed activate vestibular receptors; (3) findings showing the distinct projections of utricular and saccular afferents to different muscle groups; (4) evidence that 500 Hz bone‐conducted vibration (BCV) induces eye movements in healthy individuals; and (5) clinical evidence indicating that oVEMPs and cVEMPs assess distinct, dissociated functions.

Thus, sound and vibration selectively activate otoliths at clinical stimulus levels. Extracellular single‐unit recordings of primary vestibular afferents in anesthetized guinea pigs have shown that 500 Hz ACS and BCV almost exclusively activate otolith afferents with irregular resting discharge. In contrast, semicircular canal neurons and regular otolith neurons are rarely activated at this frequency, and only at very high stimulus intensities [10]. Curthoys and colleagues [43] has highlighted how the origin of these activated neurons has been identified through electroporation (juxtacellular injection) of neurobiotin into the irregular otolith neurons. Following this, the entire utricular and saccular maculae are extracted and processed with immunohistochemistry to stain and trace the labeled neurons. This approach allows researchers to determine not only which macula the labeled neurons originated from but also their precise location within the macula.

Several studies [15, 16, 25] consistently showed that diminished or absent VEMPs responses indicate localized vestibular dysfunction. Since these tests may be the only ones capable of objectively identifying specific pathologies of the vestibular system, they should be also included in the diagnostic work‐up of Persistent Postural‐Perceptual Dizziness (PPPD) [44] considering the possibility of vestibular pathologies that may otherwise go undetected, such as isolated utricular deficits. Currently, no instrumental test is capable of directly diagnosing PPPD, although patients with this condition may present with abnormal gait stability [45]. VEMPs, therefore, should be regarded as a necessary tool to rule out coexisting vestibular disorders. Furthermore, prior to the onset of PPPD, they may serve as a useful tool in identifying a specific triggering event, thereby enabling a more tailored and effective rehabilitative approach.

Several studies [35, 36, 38, 39, 40] support the inclusion of VEMPs in a multimodal diagnostic approach alongside tools such as the vHIT, calorics to evaluate both dynamic and static semicircular canal function and SVV in addition to ocular cervical reflex to consider both dynamic and static otolith contribution to balance. Notably, abnormal SVV values (> 2°) have been observed in over 70% of VN patients, particularly those with complete canal paresis, and have shown significant correlation with oVEMP asymmetry ratios, suggesting a predominant utricular involvement [46, 47]. The integration of these modalities has been shown to improve the characterization of vestibular deficits [26], offering clinicians not only greater diagnostic precision but also a more comprehensive understanding of peripheral vestibular disorders [48]. When combined, these tools allow for cross‐validation of findings, enhancing the reliability and clinical utility of the diagnostic process. This integrated approach is particularly valuable when traditional tests yield inconclusive results, as it supports more tailored and effective therapeutic strategies.

While most of the existing literature focuses on peripheral disorders such as VN, several studies have emphasized the importance of extending vestibular assessment to central neurological conditions. The acute phase of vestibular symptoms remains diagnostically challenging, especially in distinguishing VN from central causes such as cerebellar stroke. It is necessary to consider that approximately 25% of AVS presentations to the emergency department represent stroke etiology [49]. It was demonstrated that vHIT was the most reliable test for differentiating VN from posterior circulation stroke (PCS), though combining VEMPs and SVV contributed additional diagnostic clarity [32]. In particular, classifying AVS patients with bilaterally preserved VOR gain values (≥ 0.70) as suspected strokes has shown a diagnostic accuracy of 90%, with a sensitivity of 88% and specificity of 92% in distinguishing central from peripheral causes [47].

In fact, the use of VEMPs in the follow‐up of VN represents another step in defining post‐insult recovery. We know that a restitutio ad integrum [50] in case of VN is possible, both for the semicircular canal system and for the otolithic system.

This information is fundamental for the clinician who can plan a rehabilitation intervention tailored to the patient in time.

Developing standardized VEMPs protocols is essential for advancing both research and clinical application. Variability in stimulus type, parameters, electrode placement, and muscle activation currently hampers comparability across studies. Consistent procedures for stimulus intensity, duration, electrode setup, and EMG monitoring would improve data harmonization and reproducibility. International collaboration is crucial to establish consensus‐based standards and foster multicenter databases for stronger meta‐analyses. Beyond diagnosis, expanding the use of vestibular testing to patients with central nervous system disorders may also enhance rehabilitation strategies [51]. The present review highlights the potential benefits of including vestibular assessments in the management of central patients, particularly in designing individualized rehabilitation programs informed by detailed functional evaluations.

We acknowledge different limitations that should be considered when interpreting the findings. First, although a comprehensive search strategy was employed across major databases, publication bias cannot be excluded, particularly as only studies published in English and available in full text were included. This language restriction could have excluded relevant data published in other languages. Second, there was substantial heterogeneity in study design, population characteristics, diagnostic protocols, and VEMPs recording techniques. Variations in the type of stimulus (i.e., Air vs. Bone Conducted), stimulus parameters (i.e., Rarefaction vs. Condensation; frequency and intensity), electrode placement, response interpretation criteria, and timing of testing post‐onset limit the comparability of results and preclude meta‐analytic synthesis.

Third, many of the included studies had small sample sizes and lacked formal power analyses, which may compromise the robustness and generalizability of their findings. Furthermore, only a few studies controlled for important confounding factors such as age, gender, comorbid conditions, or medication use, all of which may influence VEMPs' responses.

Lastly, the absence of standardized criteria for defining abnormal VEMPs responses across studies complicates the interpretation of diagnostic performance and limits the ability to draw firm conclusions about their clinical utility in VN.

Another area requiring further investigation is the relationship between VEMP findings and patient‐reported outcomes. Viciana and colleagues [33] reported no significant correlation between VEMPs and measures such as the Dizziness Handicap Inventory (DHI) or the SF‐36. However, this issue remains underexplored. Future systematic reviews and meta‐analyses should examine whether VEMP abnormalities correlate with symptom severity, functional status, or recovery trajectories. It is also necessary to determine whether normalization of VEMP responses is associated with clinical improvement, and whether specific patterns of electrophysiological recovery can predict long‐term outcomes. Additionally, further research should explore the correlation between different instrumental tests. The lack of consistent association between SVV and oVEMPs may be influenced by the timing of the assessment [47]. For instance, the correlation between SVV and oVEMPs appears to be most evident in the acute phase of vestibular loss [52, 53], while SVV tends to normalize over time due to central compensation, even when oVEMP responses remain abnormal.

## 5 Conclusion

VEMPs represent a valuable component of the diagnostic battery for VN, particularly when interpreted in conjunction with other clinical and instrumental data. Standardization of protocols, integration with complementary tests, and exploration of patient‐centered outcomes are key areas for future development. Further studies are needed to clarify whether VEMPs should remain a supportive tool, as currently indicated, or be redefined as a primary test within the vestibular assessment for patients with symptoms consistent with VN.

## Conflicts of Interest

The authors declare no conflicts of interest.

## Supporting information


**Table S1:** Main characteristics of the observational studies included in the review.

**Data S1:** Search strategy used for the Scoping Review.

**Data S2:** Preferred Reporting Items for Systematic Reviews and Meta‐Analyses extension for Scoping Reviews Checklist.

## Data Availability

Data sharing not applicable to this article as no datasets were generated or analyzed during the current study.
