# Supplementary material for: Vestibular Evoked Myogenic Potentials to Diagnose Vestibular Neuritis: A Scoping Review
Source: Laryngoscope. 2025 Oct 22;136(4):1635–44. doi: 10.1002/lary.70214 (PMC12993097; doi:10.1002/lary.70214)
Supplement: Supplementary file 1 — Table S1: Main characteristics of the observational studies included in the review. [file LARY-136-1635-s003.docx]

Supporting Table 1

| **First Author, year** | **Type of VEMPs** | **Stimulus Parameters** | **Stimulus Type** | **Electrode Placement** | **Muscle Activation Method** | **Recording Device** | **Outcome Measures** |
| --- | --- | --- | --- | --- | --- | --- | --- |
| Hong, 2008 | cVEMPs | 0.1 ms duration, 95 dB, 5 per second | ACS | Active on the middle third of SCM, reference on the upper sternum, ground on the forehead | Head rotated away from the stimulated side | Nicolet Viking IV, bandpass filtered (20 Hz to 2 kHz), 100 ms analysis window, 256 stimuli averaged | p13-n23 amplitude, latencies (p13, n23), VEMPs asymmetry (VA) |
| Manzari, 2010 | oVEMPs and cVEMPs | 500-Hz tone burst (7 ms total, 1-ms rise/fall, 5-ms duration) or 1-ms square wave; 130 dB Force Level re 1 𝜇N | BCV | oVEMPs: Active electrode beneath the lower eyelid, reference electrode 2 cm below it  cVEMPs: Active electrodes on the SCM | oVEMPs: Subjects supine, looking up at a target 25° above visual straight ahead  cVEMPs: Subjects supine, lifting head from the pillow | Medelec Amplaid MK12, EMG sampled at 20 kHz, band-pass filtered (3–500 Hz), averaged | Presence/absence of VEMPs responses |
| VIciana, 2010 | cVEMPs | 129 dB SPL, Blackman gating function, 2-cycle rise/fall, 5 Hz rate, 126 trials | ACS | Positive on upper SCM, reference on lateral end of upper sternum | Head turned away from stimulated side, maintaining 50-250 μV EMG activity | SmartEP system (Intelligent Hearing Systems) with EMG amplification/filtering (20-1500 Hz) | P1 latency, N1 latency, corrected P1 N1 amplitude (compared to lab norms); SF-36 and DHI-S |
| Curthoys, 2011 | oVEMPs and cVEMPs | The ACS stimuli for oVEMPs and cVEMPs were 95 dBnHL tone  bursts of 4 ms duration. The stimulation rate  was 5 Hz. The stimulus was 500 Hz short tone bursts. | ACS+BCV | oVEMPs: EMG electrodes placed just beneath the eyes. The active EMG electrodes were placed on the cleaned skin over the infra-orbital ridges at the cen-  tre of each lower eye lid and the reference electrodes were placed 3 cm below that.  cVEMPs: EMG  electrodes were placed over the belly of the SCM and the patient  contracted the SCM by lifting their head off the pillow.. | oVEMPs: A small fixation point was positioned 25–30 deg above the  patient’s visual straight ahead.  cVEMPs: Patients contracted the SCM by lifting their head off the pillow. | The stimulator was a  Bruel and Kjaer Mini-Shaker (4810) (Naerum, Denmark), fitted with a 2 cm long machine screw (M5) terminated in a bakelite cap 1.5 cm in diameter. | The n10 component of the oVEMPs. |
| Govender, 2011 | oVEMPs and cVEMPs | AC: 500 Hz short tone burst (2 ms duration), 0.1 ms square wave click (140 dB peak SPL), 400 Hz short tone burst (2.5 ms)  BC: Lateral impulses with a minishaker (mean whole-head acceleration ~0.13g), taps delivered with an electronically triggered reflex tendon hammer  Stimulus intensities: 105–113 dB LAeq (max 140 dB peak SPL)  Repetition rate: 5 Hz for AC, 2 Hz for tap stimulation | ACS+BCV | cVEMPs: Over sternocleidomastoid (SCM) muscle  oVEMPs: Below the eye (upper electrode near orbital margin, lower ~2–3 cm below) | cVEMPs: Head lifted against gravity (supine, 30° reclined)  oVEMPs: Upward gaze (≥20° above horizontal) | EMG sampling rate: 5 kHz (cVEMPs), 10 kHz (oVEMPs)  Equipment: SIGNAL software, 1401plus laboratory interface, self-adhesive Ag/AgCl electrodes | cVEMPs: p13–n23 peak (AC), i-p1/i-n1 (ipsilateral BC), c-p1 (contralateral BC)  oVEMPs: n1–p1 (AC, forehead tap), c-n1/c-p1 (contralateral BC), i-n1 (ipsilateral BC) |
| Lin, 2011 | oVEMPs and cVEMPs | oVEMPs:  Hand-held electromechanical vibrator (V201, Ling Dynamic Systems); the input signal was a half-cycle of sine wave (1.0 ms duration)  Peak driving voltage: 8.0 V (128 dB force level)  Stimulation rate: 5 Hz  Analysis time: 50 ms  30 responses averaged per run  cVEMPs:  Short tone bursts (500 Hz)  Rise/fall time: 1 ms, plateau time: 2 ms  Intensity: 105 dB nHL (127 dB SPL)  Rarefaction polarity  Stimulation rate: 5 Hz  Analysis time: 60 ms  100 responses averaged per run | oVEMPs: BCV  cVEMPS: ACS | oVEMPs:  Active electrodes: ~1 cm below lower eyelids  Reference electrodes: 1–2 cm below active electrodes  Ground electrode: Sternum  cVEMPs:  Active electrodes: Upper half of SCM  Reference electrode: Suprasternal notch  Ground electrode: Forehead | VEMPs: Subject seated, looking upward (>2 m target, 30° visual angle)  cVEMPs: Subject seated, head rotated sideways toward one shoulder to activate the SCM | oVEMPs: Smart EP 3.90 (Intelligent Hearing Systems)  cVEMPs: EMG signals amplified and bandpass-filtered (1–1000 Hz) | oVEMPs:  Biphasic peaks: nI and pI  Measured parameters:  Latencies of nI and pI  nI-pI interval and amplitude  Asymmetry ratio: (larger amplitude – smaller amplitude) / (larger amplitude + smaller amplitude) × 100  Norms:  nI latency: 9.2 ± 0.9 ms (delayed if >11.0 ms)  Asymmetry ratio: >35% considered reduced response  cVEMPs: Biphasic peaks: p13 and n23  Measured parameters:  Latencies of p13 and n23  p13-n23 amplitude  Asymmetry ratio (>41% considered reduced response)  Norms:  p13 latency: 13.5 ± 0.8 ms (delayed if >15.1 ms) |
| Oh, 2013 | oVEMPs and cVEMPs | ACS: 1000-Hz, 5 ms duration, 50 µV amplitude, 100 stimuli at 5 Hz.  BCV: 500-Hz, 85 dB FL, 100 stimuli at 5.1 Hz.  Sampling rate: 10 kHz (oVEMPs), 5 kHz (cVEMPs).  Analysis window: oVEMPs (10 ms before to 50 ms after stimulus onset), cVEMPs (10 ms before to 60 ms after stimulus onset). | ACS+BCV | oVEMPs: Active electrodes placed 1 cm below the center of the lower eyelids, reference electrodes 2 cm below, ground electrode on the forehead.  cVEMPs: Active electrodes over the belly of the ipsilateral SCM, reference electrode on the medial clavicle, ground electrode on the sternum. | oVEMPs: Subjects lay supine, maintaining an upward gaze (25°) at a fixation point 60 cm away.  cVEMPs: Subjects lay supine, lifting their head to 30° while rotating it contralaterally to activate the SCM. | Differential amplifiers with a bandwidth of 10–2000 Hz.  ACS signals sampled at 10 kHz, BCV signals at 5 kHz.  EMG signals band-pass filtered (10–3000 Hz for cVEMPs, 10–2000 Hz for oVEMPs).  Unrectified signals averaged (n = 100). | Peak latencies: p13 and n23 for cVEMPs, n10 for oVEMPs.  Amplitude: p13-n23 for cVEMPs, n10 for oVEMPs.  Asymmetry Ratio (AR) calculated as (larger amplitude – smaller amplitude) / (larger amplitude + smaller amplitude) × 100.  Responses classified as present if peaks were reproducible across consecutive runs. |
| Walther, 2013 | oVEMPs and cVEMPs | 100 dB nHL, 7 ms duration (cVEMPs), 4/s rate (oVEMPs) | ACS | oVEMPs: infraorbital margin, forehead  cVEMPs: SCM, forehead, jugular region | Tonic contraction of SCM by raising head (cVEMPs), upward gaze (oVEMPs) | EMG with amplification and filtering (5 Hz - 800 Hz) | VEMPs amplitude and latency, with reproducibility > 90% after 100-200 stimuli |
| Adamec, 2013 | cVEMPs and oVEMPs | The clicks were delivered at an intensity of 130 dB SPL with a stimulation rate of 1 Hz. Each ear received 50 trials, repeated twice for reproducibility. | ACS | cVEMPs: The active electrode was placed on the belly of the stimulated SCM, with a reference electrode on the tendon of the same SCM.  oVEMPs: The active electrode was positioned 2 cm below the contralateral eye, with the reference electrode placed 1 cm below. | SCM (cVEMPs): Patients maintained contraction by pushing against an elastic band strapped over their forehead.  Ocular Muscles (oVEMPs): Patients gazed at the ceiling to activate the ocular muscles. | The Brain Products Brain Vision Recorder (Germany) was used for data collection, with signal analysis performed using the Brain Vision Analyzer. Signals were filtered with a bandpass filter from 5 to 1000 Hz. | oVEMPs responses were analyzed by measuring N10-P13 peak-to-peak amplitude.  Patients were classified into two groups based on their oVEMPs results at six months:  Group 1: Showed improvement in oVEMPs amplitude.  Group 2: Showed no change or worsening in oVEMPs amplitude.  A decrease of 50% in oVEMPs amplitude compared to normative values or the healthy side was considered abnormal. |
| Magliulo, 2014 | oVEMPs and cVEMPs | cVEMPs: 500-Hz logon (negative polarity), 130 dB SPL, 4 stimuli per second  oVEMPs: Handheld mini-shaker (4810; Bruel & Kjaer) applied to the forehead | oVEMPs: BCV  cVEMPs: ACS | oVEMPs:  Reference electrodes: Below the center of the lower eyelids  Exploring electrodes: 1-2 cm below the reference electrodes  Ground electrode: Forehead  cVEMPs:  Reference electrodes: Sternocleidomastoid (SCM) muscles bilaterally  Exploring electrode: Middle of the clavicle  Ground electrode: Forehead | cVEMPs: Patient lying supine, asked to lift and bend the head toward the front of the neck to activate the SCM bilaterally  oVEMPs: Patient lying supine, looking at a target placed 15°-30° backward at a distance of 2 m | cVEMPs: MK22 Amplaid electromyography unit  oVEMPs: Handheld mini-shaker (4810; Bruel & Kjaer) | cVEMPs and oVEMPs abnormalities defined by:  Absence of response  Increased latency  Decreased amplitude (p1-n1 complex for cVEMPs, N10 wave for oVEMPs) beyond 2 standard deviations of normative values  Ipsilateral analysis for cVEMPs, contralateral analysis for oVEMPs |
| Magliulo, 2014 | oVEMPs and cVEMPs | NA | NA | NA | cVEMPs were analyzed ipsilaterally, requiring sternocleidomastoid muscle activation.  oVEMPs were analyzed contralaterally due to their crossed vestibulo-ocular reflex (VOR). | MK22 Amplaid electromyography unit. | The absence of response, increased latency, or decreased amplitude beyond 2 standard deviations based on age-related normative data.  cVEMPs responses were analyzed ipsilaterally, while oVEMPs responses were assessed on the contralateral side due to the crossed VOR. |
| Nagai, 2014 | oVEMPs and cVEMPs | oVEMPs: 500 Hz, 115 dB force level, 50 stimuli at 5 Hz, analysis time of 40 ms.  cVEMPs: Rarefaction clicks (105 dB nHL, 0.5 ms), 5 stimuli per second. | oVEMPs: BCV  cVEMPs: ACS | oVEMPs: Active electrode on the inferior orbital margin, reference electrode 2 cm below, ground electrode on the para-medial forehead.  cVEMPs: Active electrode over the upper half of the SCM muscle, reference electrode on the upper edge of the sternum, ground electrode on the forehead. | oVEMPs: Subjects lie supine with a 30° upward gaze while focusing on a target positioned in the upper 30° of the visual field.  cVEMPs: Subjects lie supine and continuously raise their head to activate the SCM. | oVEMPs: Signals amplified and band-pass filtered between 20 and 2000 Hz, responses averaged over 50 ms.  cVEMPs: Surface EMG electrodes amplify signals from the SCM muscle, band-pass filtered and sampled accordingly. | oVEMPs: Amplitude of nI (measured from baseline to peak), asymmetry ratio (AR) calculated as {(larger nI – smaller nI)/(larger nI + smaller nI)} × 100.  cVEMPs: p13-n23 amplitude ratio calculated between the lesion side and the healthy side. Abnormal VEMPs value is defined by a ratio of < 0.5.  Normal oVEMPs asymmetry ratio (AR) range: 21.7 ± 14.0, upper limit set at 49.7. |
| Skoric, 2017 | oVEMPs and cVEMPs | oVEMPs: 1 ms acoustic click, 130 dB SPL, stimulation frequency of 1 Hz, series of 50 stimuli per ear for reproducibility.  cVEMPs: 1 ms acoustic click, 130 dB SPL, stimulation frequency of 1 Hz, series of 50 stimuli per ear for reproducibility. | ACS | oVEMPs: Surface electrodes placed 2 cm below the contralateral eye to the stimulated ear.  cVEMPs: Surface electrodes placed on the belly and tendon of the SCM muscle on the side ipsilateral to the stimulated ear. | oVEMPs: Subjects instructed to gaze upward to activate ocular muscles.  cVEMPs: Subjects instructed to slightly move their head away from the chair and push it against an elastic band around the forehead to activate the SCM. | oVEMPs & cVEMPs: Brain Products Brain Vision Recorder and Brain Vision Analyzer (Brain Products GmbH, Munich, Germany). | oVEMPs: Peak-to-peak ocular muscle (OM) amplitude (OL N10-P13, OR N10-P13), asymmetry ratio (AR) calculated as:  AR = (ARight – ALeft)/(ARight + ALeft) and considered pathological if ≥33%.  cVEMPs: Normalized SCM amplitude (SCMR CorAmp, SCML CorAmp), asymmetry ratio (AR) calculated as:  AR = (ARight – ALeft)/(ARight + ALeft) and considered pathological if ≥33%. vHIT: RL and LL gain at 60 ms, presence  of covert and overt saccades and gain asymmetry; RA, LP, LA, RP  slope and presence of covert and overt saccades. LL and RL gains at 60 ms were considered pathological if <0.75. |
| Cherchi, 2019 | oVEMPs and cVEMPs | NA | NA | NA | NA | Bio-Logic Navpro system for oVEMPs; | Altereted/ not alterated |
| Tripathi, 2020 | cVEMPs | Stimulus Parameters  Frequency: 500 Hz  Stimulus Type: Tone bursts  Duration: 0.2 ms per burst  Intensity: 105 dB nHL and 50 dB nHL  Number of stimuli: 200 stimuli in total | ACS | Active (positive) electrode: Middle one-third of the ipsilateral sternocleidomastoid (SCM) muscle to be tested  Reference (inactive) electrode: On the sternal head of the SCM muscle  Ground electrode: On the forehead | The patient was seated with the head turned to the contralateral side from the ear being tested, which facilitates the activation of the SCM muscle during cVEMPs testing. | The recordings were performed using Epic Plus equipment, manufactured by Labat International. The software provided the results in graphical form. | Amplitude: Measured in microvolts and recorded on the Y-axis  Latency: Measured in milliseconds and recorded on the X-axis  Key Waves:  P13: First positive peak, downward deflection, recorded at 13 ms  N23: First negative peak, recorded at 23 ms  Summation Potentials: Three consecutive recordings were made to confirm the presence of cVEMPs. |
|  |  |  |  |  |  |  |  |
| Calic, 2020 | oVEMPs and cVEMPs | AC Stimulus:  Duration: 0.1 ms clicks  Intensity: 140 dB peak sound-pressure level  BC Stimulus:  Pulse duration: 1.0 ms condensation-polarity  Driving voltage: 20 V peak-amplitude  Stimulus was applied using a Bruel and Kjaer 4810 minishaker. | oVEMPs: BCV  cVEMPs: ACS | NA | NA | NA | Asymmetry Ratios (AR) were used rather than amplitudes to assess unilateral otolith dysfunction.  Jongkees-Formula was used to calculate AR.  AR values were compared against laboratory control data:  Upper limit of normal:  30.2% for AC-cVEMPs  38.1% for BC-oVEMPs  For participants with unilaterally absent responses, an amplitude of zero was assigned, resulting in an AR of 100%.  Bilaterally absent VEMPs were excluded from the analysis. |
| Manzari, 2021 | oVEMPs, specifically the n10 component | The stimuli are 500 Hz BCV and ACS. | ACS+BCV | Active electrode: Placed beneath the eyes, on the skin.  Reference electrode: Also beneath the eyes. | The response is elicited by activation of the inferior oblique eye muscles, which are stretched by the vestibular stimulation. | NA | n10 |
| Kabis, 2022 | oVEMPs and cVEMPs | Stimulus intensity: 90 dB nHL (127 dB SPL)  Stimulus duration: 0.1 ms  Number of stimuli: 250 stimuli presented for both oVEMPs and cVEMPs tests. | ACS | oVEMPs: Electrodes were placed beneath the contralateral eye.  cVEMPs: Electrodes were placed over the SCM. | cVEMPs: Patients were instructed to turn their head towards the contralateral shoulder and maintain a constant muscle contraction (EMG ≥ 50–200 μV) throughout the test.  oVEMPs: Patients were asked to look upward while seated, aiming at an object forming a 30° angle with the horizontal axis. | NA | oVEMPs:  Peak latency, peak-to-peak amplitude, and asymmetry ratio (AR) were calculated for the n1 and p1 peaks.  Abnormal AR: Above 40% or below −40% was considered abnormal.  cVEMPs:  AR values above 30% or below −30% were considered abnormal. |

Supporting Table 1: Main characteristics of the observational studies included in the review.
