# Supplementary material for: Vestibular Evoked Myogenic Potentials to Diagnose Vestibular Neuritis: A Scoping Review
Source: Laryngoscope. 2025 Oct 22;136(4):1635–44. doi: 10.1002/lary.70214 (PMC12993097; doi:10.1002/lary.70214)
Supplement: Supplementary file 2 — Data S1: Search strategy used for the Scoping Review. [file LARY-136-1635-s001.docx]

Supporting Material 1

Search terms were modified for each database and appropriate subheadings were used for each database searched. The following strings were used in the respective databases:

PubMed: (("vestibular neuritis"[MeSH Terms] OR "vestibular neuritis" OR "vestibular neuronitis") AND ("vestibular evoked myogenic potentials"[MeSH Terms] OR "VEMP" OR "cVEMP" OR "oVEMP" OR "vestibular myogenic potentials") AND ("diagnosis"[MeSH Terms] OR "diagnostic" OR "evaluation")).

Scopus: (TITLE-ABS-KEY("vestibular neuritis" OR "vestibular neuronitis") AND TITLE-ABS-KEY("VEMP" OR "cVEMP" OR "oVEMP" OR "vestibular evoked myogenic potentials") AND TITLE-ABS-KEY("diagnosis" OR "diagnostic" OR "evaluation")).

Cochrane: ("vestibular neuritis" OR "vestibular neuronitis") AND ("VEMP" OR "cVEMP" OR "oVEMP" OR "vestibular evoked myogenic potentials") AND ("diagnosis" OR "diagnostic").
